# The Evolving Contribution of Mass Spectrometry to Integrative Structural Biology

**DOI:** 10.1007/s13361-016-1382-4

**Published:** 2016-04-07

**Authors:** Marco Faini, Florian Stengel, Ruedi Aebersold

**Affiliations:** Department of Biology, Institute of Molecular Systems Biology, ETH Zürich, 8093 Zürich, Switzerland; Department of Biology, University of Konstanz, 78457 Konstanz, Germany; Faculty of Science, University of Zürich, Zürich, Switzerland

**Keywords:** Structural mass spectrometry, Protein complexes, Hybrid modeling, Integrative structural biology, Affinity purification MS, Cross-linking mass spectrometry, XL-MS, CX-MS, Native MS

## Abstract

Protein complexes are key catalysts and regulators for the majority of cellular processes. Unveiling their assembly and structure is essential to understanding their function and mechanism of action. Although conventional structural techniques such as X-ray crystallography and NMR have solved the structure of important protein complexes, they cannot consistently deal with dynamic and heterogeneous assemblies, limiting their applications to small scale experiments. A novel methodological paradigm, integrative structural biology, aims at overcoming such limitations by combining complementary data sources into a comprehensive structural model. Recent applications have shown that a range of mass spectrometry (MS) techniques are able to generate interaction and spatial restraints (cross-linking MS) information on native complexes or to study the stoichiometry and connectivity of entire assemblies (native MS) rapidly, reliably, and from small amounts of substrate. Although these techniques by themselves do not solve structures, they do provide invaluable structural information and are thus ideally suited to contribute to integrative modeling efforts. The group of Brian Chait has made seminal contributions in the use of mass spectrometric techniques to study protein complexes. In this perspective, we honor the contributions of the Chait group and discuss concepts and milestones of integrative structural biology. We also review recent examples of integration of structural MS techniques with an emphasis on cross-linking MS. We then speculate on future MS applications that would unravel the dynamic nature of protein complexes upon diverse cellular states.

**Graphical Abstract:**
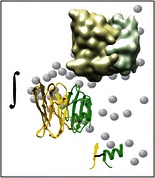
ᅟ

ᅟ

## MS and the Structural Biology of Protein Complexes

Mass spectrometry (MS) is an analytical technique that has found wide application in biological research. The development of instrumentation and experimental workflows targeted at the identification and quantification of ever larger sets of proteins has revolutionized our understanding of the expressed proteome and how it is related to the diverse functional states of the cell [1]. The advent of faster mass spectrometers and the combination of MS/MS with affinity purification (AP-MS) allowed the isolation and identification of a protein of interest (bait) in association with its physiological interactors [2]. While AP-MS results can hint at the concerted function of a bait and its interactors, the technique revealed its potential when applied to systematically map the interactome of an organism. Combining protein identifications from multiple baits exposed a network of interactions within the cell and demonstrated a generic modular organization of the proteome first in yeast cells [3–5] and, more recently, in human cell lines [6, 7]. However, because the results of AP-MS studies report the sum of proteins associated with the bait protein, in cases in which the bait protein is associated with several complexes the data do not actually determine the composition of a functional protein complex. As protein complexes are the functional modules of the cell, studying their assembly and structure is fundamental for a functional understanding of the cellular inner workings and requires analytical methods that go beyond the capabilities of AP-MS.

## Structural Mass Spectrometry Techniques

Mass spectrometry has contributed to our understanding of protein complexes for many years. The versatility and sensitivity of mass spectrometers spurred the development of a variety of techniques. The group of Brian Chait significantly contributed to the inception of these technical developments and realized several pioneering advances. For example, limited proteolysis coupled to MS [8, 9] and hydrogen-deuterium exchange MS (HDX-MS) [10] have been employed to characterize protein–protein interfaces and monitor conformational changes. More recently, native MS and ion mobility MS (IM-MS) have emerged to study the assembly and disassembly pathway of whole complexes, the connectivity between subunits, and their stoichiometry [11–14]. For a detailed review of these techniques we refer to [15, 16].

Among the structural techniques employing MS, cross-linking mass spectrometry (XL-MS) has probably gained the most interest in the community of structural biologists because of its relative simplicity of implementation, reproducibility, and high information content of the obtained distance restraints. In XL-MS, the chemical linking of amino acid residues within a native protein complex are combined with the direct identification of cross-linked peptides by tandem MS. By identifying cross-links between amino acid residues that are nearby in space, XL-MS can prove the physical proximity of protein complex subunits, determine their topological arrangement, and indicate the precise location where protein domains interface. Furthermore, the length of the cross-linker defines an upper bound of the distance between the cross-linked residues that proves useful for orienting and docking known atomic structures. For a detailed account of the recent innovations in XL-MS, we refer to [17–19].

## Conventional Structural Techniques

Structural biology has been traditionally driven by high-resolution techniques such as X-ray crystallography and nuclear magnetic resonance (NMR). For many years, these techniques produced structures of isolated proteins and dimers [20]. Once it became evident that protein complexes are responsible for fundamental cellular functions, the interest of structural biologists shifted towards characterizing larger assemblies of multiple subunits. Although many important protein complex structures have been solved by traditional structural techniques, their requirement for large sample amounts and high purity limited their application to large scale experiments.

Recently, cryo-electron microscopy (cryo-EM) emerged as an alternative method to tackle the structure of protein complexes. In most cases, cryo-EM is performed on isolated protein complexes frozen in a thin layer of amorphous ice and, therefore, does not need sample crystallization. Furthermore, cryo-EM sample preparation requires a fraction of the sample amount needed for X-ray crystallography. Recent developments in direct electron detectors and image processing software have improved the attainable resolution and extended the molecular mass range of the technique [21, 22]. Although the number of solved structures of protein complexes has steadily increased, many complexes proved recalcitrant to any single structural method, especially those where sample amount is limited and compositional or structural heterogeneity is expected. Solving the structure of such complexes increasingly requires integrating several complementary techniques. This approach is often referred to as hybrid modeling or integrative structural biology.

**Figure 1:**
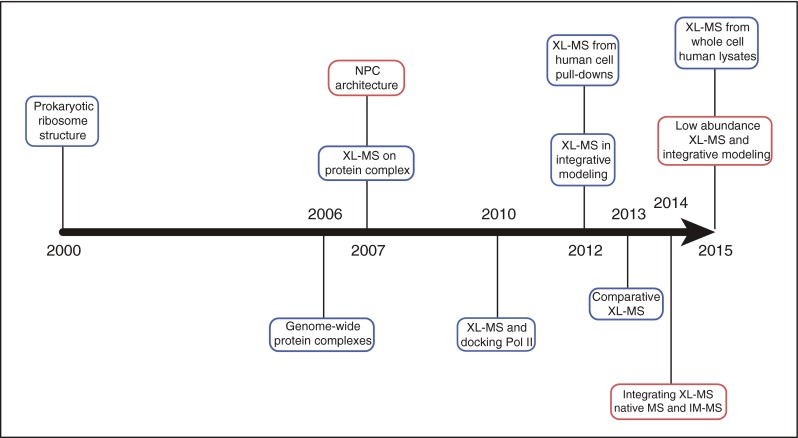
Milestones in XL-MS of protein complexes and integrative structural biology. The timeline illustrates important innovations in XL-MS of protein complexes along with fundamental milestones in the study of protein complexes and integrative structural biology. The contributions of Brain Chait’s group are highlighted in red. The events refer to: 2000 [23]; 2006 [4, 5]; 2007 [24–26]; 2010 [27]; 2012 [28, 29]; 2013 [30]; 2014 [31]; 2015 [32, 33]

In this perspective article, we describe the principles of integrative structural biology and discuss the evolving contributions of mass spectrometry to this emerging field, with a particular emphasis on XL-MS. We then discuss how innovations in structural mass spectrometry will facilitate and support the structural characterization of protein complexes in a close to native environment and the study of their dynamics in different cellular states. For many years, Brian Chait and his group have been at the forefront of such innovations and have contributed seminal work to virtually every structural technique in mass spectrometry. Here we mention some of his major discoveries in the context of this exciting and rapidly progressing field of research. We also summarize the contributions of his group along with major milestones of structural mass spectrometry in Figure 1.

## Integrative Structural Biology

Integrative structural biology is a discipline concerned with the collection of a diverse set of interaction and structural data, and their integration into an organized computational framework with the aim of proposing comprehensive structural models. By combining data from complementary techniques, it can overcome technical limitations of individual techniques and describe systems spanning wide ranges of size and complexity that may be beyond the reach of each technique. The integrative approach is particularly suitable to large, heterogeneous, and flexible complexes and has the potential to increase the throughput of structural studies [34, 35]. Integrative modeling can incorporate heterogeneous sources of data such as genetic and biochemical data (genetic mutations, yeast-two-hybrid, AP-MS, XL-MS, HDX-MS), spectroscopic data (NMR, EPR, FRET), atomic models (X-ray crystallography), density maps (EM, SAXS), as well as physical constraints (cross-sectional areas, excluded volumes).

**Figure 2:**
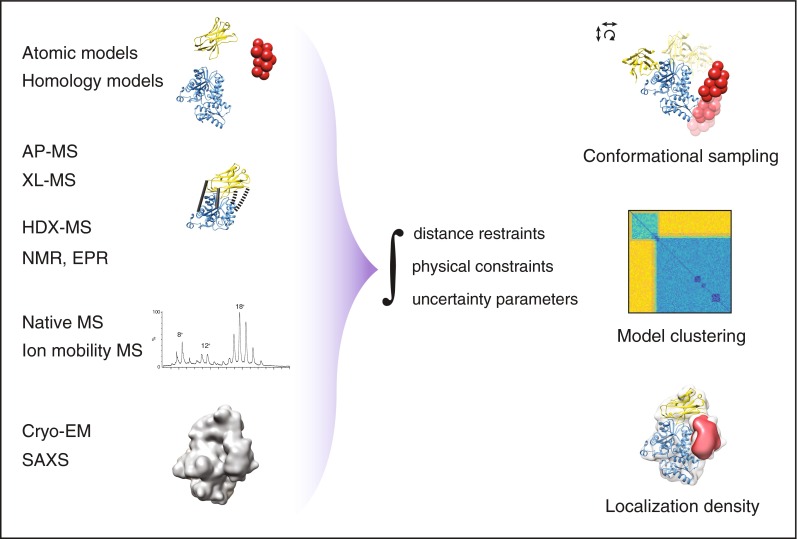
A general integrative structural biology workflow. First, data such as protein sequences, atomic models (in blue and yellow), and restraints (e.g., from AP-MS, XL-MS, HDX-MS, NMR, and EPR) are collected, together with density (in gray), (cryo-EM), or envelope data (SAXS). If atomic models are not available, protein densities can be represented by homology models or by beads as placeholders (red spheres). Next, the data is integrated with distance restraints, the densities, and physical constraints into a comprehensive scoring function. The data ensemble is then shifted and rotated (conformational sampling) and each resulting model is scored according to the optimal satisfaction of restraints. The models are then clustered by their structural similarity and a consensus model derived from the cluster is represented as a localization density

The typical integrative modeling workflow consists of multiple steps (Figure 2). First, structural information of components of an assembly are gathered. This information includes the amino acid sequence, atomic models, comparative models, or electron density maps. Regions of unknown structure are represented computationally, for example, by beads that act as placeholders. The size of the beads is variable and is matched to the occupied volume of single amino acids, secondary structural elements, or entire protein domains. These components are then combined with distance restraints and physical constraints and considered together in a comprehensive scoring function. Sampling of this function by translating and rotating each component results in a set of models with different degree of agreement with the input data. Then, a validation step evaluates the set of models according to their satisfaction of restraints. Lastly, models are clustered according to their structural similarity and a consensus model, usually consisting of an ensemble of models, is proposed. The systematic nature of the workflow facilitates its sharing with the research community and subsequent update with new data. Similar workflows have been implemented in different software packages [34, 36, 37].

## The Architecture of the Nuclear Pore Complex

One of the earliest examples of integrative structural modeling originated from the collaborative effort of the groups of Michael Rout, Brian Chait, contributing essential mass-spectrometry data, and Andrej Sali. The work represents a milestone of modern structural biology: the molecular architecture of the yeast nuclear pore complex (NPC) [24, 25]. The NPC is one of the largest assemblies of the cell; due to its size and complexity, it lies far beyond the realm of any single structural technique. The groups set out, in a collaborative effort, to collect diverse and orthogonal datasets. First, a low resolution cryo-EM density model provided the overall shape of the NPC and hinted at the internal pore symmetries (eight spokes for each of the two nuclear and cytoplasmic sides). An extensive experimental effort identified all nuclear pore proteins and their interactors by affinity purifications followed by in-gel proteolysis and MALDI MS [38]. Similarly, the stoichiometry of the interactors was determined by quantitative immunoblotting of the same tagged proteins. Then, sedimentation analysis on sucrose gradients of individual proteins provided their approximate size and shape, which were then used to build, together with domain predictions, in silico bead models for each protein. Because the NPC spans hundreds of nanometers, it was relevant to know the location of the individual subunits. To this end, immuno-gold labeling followed by 2D electron microscopy provided the approximate location of the tagged proteins in the context of the whole pore.

To integrate the heterogeneous datasets, the authors first developed an algorithm that inferred proximity and connectivity maps within each subcomplex from AP-MS data. This dataset provided the richest set of restraints for the determination of the protein configurations within the pore. Then, each protein was represented as one or more beads depending on their approximate shape and expected domain folds. A scoring function was devised integrating the positions of each protein and the distance restraints from the other experiments. The computational framework was implemented in the open source modeling suite Integrative Modeling Platform (IMP) [34]. Starting from a large set of random configurations, the algorithm applied translations and rotations to the modeled proteins and minimized the scoring function (i.e., testing protein configurations that would satisfy the largest number of restraints).

From this extensive experimental and computational effort, the authors could assign a localization probability for each protein, suitable to determine their relative position within the pore. In the nuclear pore architecture, the authors could localize proteins with folds typical of coat proteins (clathrin, COPI, and COPII) close to the nuclear membrane confirming the evolutionary relatedness of membrane bending systems. Furthermore, the arrangement of pore spokes in vertical columns suggested that the underlying principle of assembly is driven by the hierarchical repetition of structural modules originated by gene duplication [25].

## The Evolving Contribution of MS to Integrative Structural Biology

Structural techniques in mass spectrometry cover a wide range of applications. Bottom-up workflows, where the analytes are generally peptides, can cope well with sample complexity. In the case of AP-MS, a few to hundreds of interactors can be identified in a single experiment. To date, XL-MS was successfully applied to assemblies with one to more than 80 subunits [39]. XL-MS provides stable, covalent linkage information between residues in close spatial proximity, but the amino acid coverage is generally low. Despite this limitation, XL-MS can uniquely provide the relative orientation of two subunits, facilitating the modeling of protein complex assemblies from individual components. HDX-MS is well suited to the investigation of conformational changes, particularly when induced by ligand binding or post-translational modifications. HDX-MS has an advantage over other techniques as it can potentially monitor the solvent exposure of every amino acid. HDX-MS is the method of choice when binding interfaces between subunits are to be sought. In contrast to bottom up approaches, ion-mobility MS and native MS require higher sample homogeneity and have limited capacity for multiplexing, but they can cope with large macromolecular complexes in their intact state. Such gas-phase techniques can determine the stoichiometry of protein complexes and the affinity and exchange rate of a complex subunit. Additionally, the generation of subcomplexes in solution or in the gas-phase can provide hints on the complex topology. Ion mobility MS and native MS are generally applied when a thorough analysis of oligomeric state distribution is desired. The unrivalled sensitivity of modern mass spectrometers handles low sample amounts and their high mass accuracy often results in a high identification specificity. Given their versatility and sensitivity, structural MS techniques are therefore ideally suited to contribute to integrative modeling efforts.

In the following, we summarize some recent developments of XL-MS and selected examples of integration of diverse MS techniques. Many of these examples build on the pioneering work achieved by the Chait group. We apologize to the many important contributions that we can unfortunately not discuss due to space limitations.

## XL-MS Towards Integrative Modeling

One of the first reports of XL-MS in the structural field was the study of the interaction between RNA polymerase II (Pol II) and the TFIIF complex [27]. The authors identified 253 cross-links between subunits and this large dataset permitted the manual positioning and orientation of the TFIIF relatively to Pol II. The proposed model for the interaction extended our understanding of transcription initiation.

Manual docking of proteins can, in some cases, lead to inaccurate models. An attempt to automate protein interface docking and exhaustively sample the orientational space was applied to the interaction of the proteasomal lid with the core proteasome particle [28]. The authors gathered multiple structural sources: a 8.4 Å resolution cryo-EM density map of the complete 26S proteasome, 15 inter-subunit cross-links between the lid and the hexameric AAA-ATPase subcomplex, direct interactions derived by previous proteomic studies, and comparative models for the individual lid subunits. To simplify the computational complexity, they represented the data as a multi-scale model in the IMP software. First they created a coarse-grained model where subunit fragments of 50 residues were depicted by a single bead. Beads would then be arranged as to mimic known atomic models or linked in flexible chains if no model was known. The EM density map defined the overall shape of the assembly and the direct interactions were used to define proximity and connectivity within the subcomplex. XL-MS restraints were only applied to coarsely define the upper distance bounds between subunits but not their orientations. After a selection of the most probable subunit location, the authors defined a finer, atomistic representation that simultaneously fitted alternative comparative models. The ensemble of 5 × 10^5^ models was then clustered by subunit location and then assessed using the XL-MS distance restraints. The model closely matched the cryo-EM reconstruction of the proteasome regulatory particle [40]. This work highlighted that XL-MS data, differently from AP-MS data, can provide distance upper-bounds and orientational information, greatly facilitating docking protocols [41]. Since then, XL-MS has been employed in multiple integrative modeling protocols [42–45].

## Expanding the Target Space of XL-MS

Although lysine residues are the most common target of cross-linking experiments, they only represent about 5% of all residues in proteins. Several groups have, therefore, developed cross-linkers that can react with other amino acids [18, 19]. One of the first applications from Kruppa and Novak [46] showed that the EDC cross-linker could conjugate an acidic residue (aspartic acid or glutamic acid) to a nearby lysine forming zero-length cross-links (conjugation of two amino acidic residues). Recently, the Chait group applied EDC to the extensively studied yeast Nup84 nucleoporin [47]. They speculated that the combination of conventional DSS cross-linking with EDC cross-linking would yield orthogonal 3D restraints between the subunits of the complex. Integrating the expanded set of restraints with known atomic models and cryo-EM density maps resulted in a detailed pseudo-atomistic model of the Nup84 complex. The proposed model was later confirmed by the crystal structure of the Nup84 complex [48]. An increase in sample complexity and the scale of XL-MS experiments necessarily requires automated analysis software. Many packages have been developed to extract and evaluate the significance of cross-linked peptides from tandem MS spectra. A large diversity of software solutions can process data obtained from isotopically coded cross-linkers [49–51], cleavable cross-linker [32, 52], as well as generic cross-linkers [49, 53].

## Integration of Multiple Structural MS Techniques

Many structural mass spectrometry techniques provide orthogonal information and can thus be combined to propose integrative structural models [54]. For example the interaction of KaiB and KaiC, components of the cyanobacterial circadian clock, was recently studied by a combination of native MS and HDX-MS [55]. First the stoichiometry of KaiB and KaiC was studied by native MS. By comparing spectra obtained from the proteins in isolation and in complex the authors could prove the transition of KaiB from monomeric to the hexameric state upon complex formation with KaiC. They then performed HDX-MS of KaiB and KaiC, first in isolation and then in complex, describing the structural changes upon interaction and defining the binding interfaces. The HDX interaction data was then submitted to the HADDOCK server to dock the proteins and the results were compared with previously described IM-MS data of the complex. Another recent report highlighted the versatility of MS techniques for the structural characterization of protein complexes. Here, native MS, IM-MS and XL-MS were combined in a generic setting and elucidated the structural organization of the yeast proteasomal lid as proof-of-principle [31]. Here, data-dependent analysis of the complexes identified their subunit composition and native MS identified the overall stoichiometry and connectivity of the intact complex and subcomplexes thereof. Orthogonal restraints derived by XL-MS confirmed many of these interactions and provided additional spatial restraints between subunits and connectivity information with peptide resolution, while IM-MS contributed rotationally averaged cross-sectional areas. The three types of information were integrated in a hybrid model that confirmed the location of subunits found in the proteasome EM density map.

**Figure 3:**
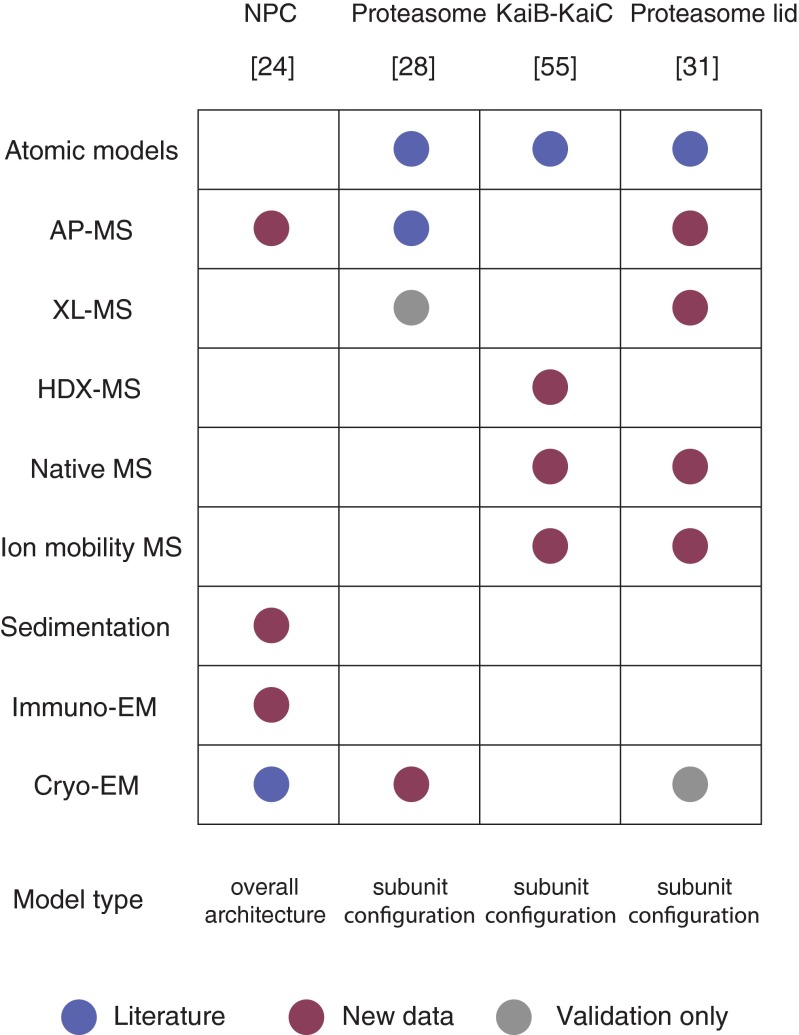
Comparison of structural methods employed in notable integrative models. The table lists integrative models described in this review, along with the structural techniques employed. Data gathered from the literature is marked in blue. Novel data is marked in brown, and data used only to validate the model is marked in gray. The modeling of the NPC complex could propose a localization density and architecture of the pore but no accurate orientation between subunits. The other studies proposed models that included the stoichiometry and the subunit configuration

In summary, structural techniques in mass spectrometry, when combined in an integrative framework, can elucidate the assembly and structural arrangement of macromolecular machines. Furthermore, the sensitivity of MS methods allows application to cases where only a limited amount of sample can be obtained or when the system is structurally heterogeneous. In those cases, MS approaches and integrative modeling can propose architectural and mechanistic models (Figure 3).

## Perspective: Towards Structures of Endogenous Complexes

Mainstream structural techniques have traditionally required large sample amounts and high sample purity. To fulfill these requirements, researchers often resorted to the expression of recombinant complex subunits of interest, first in prokaryotic and then in eukaryotic hosts [56, 57]. While it would be desirable to attain structural characterization from endogenously expressed complexes, most structural techniques cannot currently cope with the limited sample amounts and the expected compositional heterogeneity. The relatively few exceptions often involve abundant complexes or require cumbersome experimental procedures [58].

Conversely, mass spectrometric applications have the potential to accurately and sensitively probe protein complexes at the typical sample scales of cell biology experiments. For example, a pioneering study could obtain XL-MS distance restraints from complexes expressed at endogenous levels, purified by affinity-tag from human cell lines [29]. The cross-linking restraints were then integrated in the ROSETTA software to propose models for the interactions between protein phosphatase 2A (PP2A) regulatory proteins and the interaction between the TRiC/CCT complex with its substrate 2ABG. Recently, an exciting report from the Chait group proved that cross-linking can yield structural information from minute sample amounts of complexes and from proteins expressed from their endogenous locus [33]. The group first developed a custom nanobody against the commonly used GFP tag. The nanobody was engineered to contain no lysines so that cross-linking could be carried out avoiding conjugation of the complex to the affinity matrix. As a proof of principle they purified the yeast exosome. Cross-links were combined with atomic structures into integrative models and the models predicted the mutually exclusive positioning of the Rpr6-LPR1 heterodimer and Ski7 in different cell compartments. Additionally, they challenged the method on the low abundant APC/C complex (10–100 copies per cell) identifying cross-links that could recapitulate the previously published cryo-EM structure [59]. Lastly, the group was even able to obtain cross-links from GFP-tagged Beclin 1-associated complexes purified from a single mouse liver. As such, the method offers the potential to be expanded to a genome-wide scale, taking advantage of the extensive collection of GFP-tagged strains [60]. A similar method was applied by the Vermeulen group who could purify and cross-link several chromatin complexes from human cells in culture [61]. Their protocol included experimental and computational innovations to reduce common contaminants of chromatin complexes and to improve the scoring of recurrent cross-links between sample analyses. Interestingly, they could compare cross-links from the MCM complex obtained from two different cell cycle phases. While it was previously speculated that the topology of MCM complexes would change dramatically upon loading onto replication forks, the structural data confirmed an invariant arrangement, in agreement with interaction studies by AP-MS.

The recent applications of XL-MS technology to natively expressed complexes both from yeast and mammalian cells opens up the possibility to study the structure of protein complexes in the context of their physiological interactors and regulators. Another emerging technique that could be applied to endogenous complexes is cryo-EM. Cryo-EM can obtain high resolution reconstructions from small sample amounts and can tolerate limited structural heterogeneity [21, 22]. The integration of such sensitive and powerful techniques would favor the generation of structural models that more comprehensively sample the physiological interaction space of protein complexes [62].

## Perspective: Structural Dynamics of Protein Complexes

Insights into the function of many protein complexes would be increased if we were able to understand their changes in different cellular states (i.e., their dynamics). Comparing different states using XL-MS requires the ability to accurately quantify the cross-linked peptides. The group of Carol Robinson pioneered this endeavor by establishing comparative XL-MS to dissect the conformational changes of the F-ATPase upon in vitro dephosphorylation [30]. A similar approach was applied to the remodeling of the proteasome lid [63] and the structural rearrangements of Cul1 complexes upon Nedd8 binding [64]. While the previous attempts relied on manual annotation and quantitation of spectra of the cross-linked peptides, an automated software based on xQuest-xProphet was recently described [65].

We think that quantitative cross-linking will expand the structural studies of conformational changes induced by protein binding and protein modifications and reveal the detailed mechanisms of large macromolecular complexes.

## Perspective: Protein Complexes in Their Cellular Milieu

A notable limitation of the previously mentioned approaches is the requirement for sample enrichment, most commonly achieved by affinity purification of tagged proteins. Although most proteomic applications of mass spectrometry can consistently explore the complexity of the cell components, XL-MS has, until now, not achieved a similar extensive coverage. Whereas previous applications in *E. coli* anticipated the experimental feasibility [66], a significant step in this direction was recently described by the Heck group. In Liu et al. [32] a human cell lysate was incubated with a cleavable cross-linker followed by a sequential fragmentation strategy (CID followed by ETD). Using a gas-phase cleavable cross-linker has the advantage of overcoming a common burden of large-scale cross-linking: the quadratic expansion of the peptide database size [49]. Overall, this strategy identified 1665 intra-protein cross-links and 514 cross-links between different proteins. Although most of the cross-linked proteins were among the most abundant in the cell, the approach represents an important step for future proteome-wide applications of structural mass spectrometry.

In summary, we envisage that the combination of recent experimental developments with powerful multiplexing capability will open up the exploration of endogenous protein complexes and the study of their dynamics and interactions upon different cellular states. Such analyses are now also generally supported by the development of a software tool that accurately quantifies the cross-links generated from complexes in different states, thus identifying subtle structural and compositional differences [65]. Furthermore, improvements in sensitivity and in cross-linking chemistry would greatly benefit the study of complexes by XL-MS with minimal disturbance to their physiological environment. The development of automated quantitative workflows will be essential for any functional study applied to protein complexes. Improvements in sensitivity of gas-phase MS techniques would favor their widespread application and benefit our understanding of protein complex dynamics. Finally and further in the future, the combination of multiple MS techniques in the framework of integrative structural biology will increasingly contribute to disentangling the structural organization of the proteome.
